# Potential Increase in Known and Emerging Biotoxins in Marine Ecosystem Due to Climate Change and Subsequent Health Issues

**DOI:** 10.3390/foods15122103

**Published:** 2026-06-11

**Authors:** Pierina Visciano

**Affiliations:** Department of Bioscience and Technology for Food, Agriculture and Environment, University of Teramo, Via R. Balzarini 1, 64100 Teramo, Italy; pvisciano@unite.it

**Keywords:** global warming, eutrophication, phycotoxins, ciguatoxins, tetrodotoxins

## Abstract

Climate change is intensifying the release and dispersion of various hazardous chemicals into marine ecosystems, such as algal biotoxins, heavy metals, persistent organic pollutants, and agricultural and industrial wastes. Eutrophication and global warming are responsible for the increase in known and emerging marine biotoxins, such as brevetoxins, palytoxins, pinnatoxins, and cyclic imines. Furthermore, tetrodotoxins and ciguatoxins, which are primarily found in tropical regions, have recently been identified in fish and bivalve molluscs from temperate areas where they had never been previously reported. These toxicants can accumulate in seafood and enter the human food chain, posing a public health concern. This review describes the interrelationship between climate change and its impact on marine organisms and human health, as well as the environment. It recommends integrating a broad range of scientific knowledge, reviewing regulatory policies, and proactively managing public health to counter these environmental threats.

## 1. Introduction

**Figure 1:**
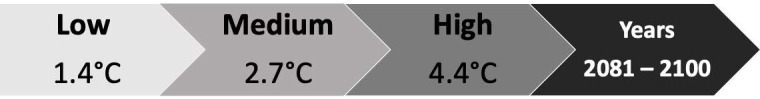
Estimated increase in global surface temperature due to greenhouse gas levels.

Climate change is a complex phenomenon resulting from both natural processes and human activities. The Intergovernmental Panel on Climate Change (IPCC) aims to proactively respond to climate-related emergencies by utilizing risk indices to assess exposure and vulnerability in various regions worldwide [1]. According to IPCC, climate change has caused widespread and rapid alterations in the atmosphere, seas and oceans, cryosphere and biosphere, with an increase in global surface temperature between 0.95 °C and 1.20 °C in the period 2011–2020 compared to the period 1850–1900. Global warming will continue to increase and is highly likely to reach or exceed 1.5 °C due to low or high greenhouse gas (GHG) emissions. Figure 1 illustrates the three potential scenarios based on GHG emission levels, leading to a median global warming of 2.8 °C by 2100. The largest share of GHG emissions comes from CO_2_, due to fossil fuel combustion and industrial processes, followed by methane. In 2019, 78% of GHG emissions derived from the energy, industry, transport, and buildings sectors, while the remaining 22% came from the agriculture, forestry and other land uses [2].

Many problems are potentially exacerbated by climate change and can impact food and feed safety, as well as plant, animal and human health, due to new and/or re-emerging diseases and zoonoses, new crop and animal pests, spread of invasive alien species, etc. Climate change can directly affect the life cycle of infectious agents, their vectors and hosts, giving rise to an increase in diseases caused by vectors (ticks, mosquitoes, sand flies, rodents, etc.) or zoonoses transmitted by air, water and food [3].

Further impacts of climate change, such as water scarcity and fertility loss, can reduce agricultural yields. In drought-affected regions of the world, agriculture may rely on untreated wastewater, which can be contaminated with microorganisms, including antimicrobial-resistant strains, and chemical pollutants. Conversely, heavy rainfall during thunderstorms can increase runoff levels in rivers and lakes, resulting in the release of animal waste, pollutants, and other materials into water resources. Severe flooding can even overload the wastewater treatment plants compromising their functioning [4,5].

The EFSA current strategy is based on the CLimate change and Emerging risks for Food SAfety (CLEFSA) project, which focuses on improving scientific knowledge across all disciplines, with the aim of providing a better understanding of the combined risks posed by global warming and climate change. Global warming is altering plant growth and biodiversity, while crop pests and diseases native to subtropical regions are moving into new geographic areas. The high temperatures that have reached the Arctic regions have caused the revitalization of bacteria and viruses called zombie pathogens, which survived for centuries in the frozen lands and are now revived thanks to the melting of the glaciers [6,7]. Similarly, many pollutants that have long been sequestered in soil and ice are being released into the environment due to the thawing of permafrost [8]. Furthermore, climate change has caused the increase and spread of biotoxins typically classified into marine (e.g., phycotoxins), fungal (e.g., mycotoxins), and plant categories (e.g., alkaloids and ricin), each of which causes specific clinical manifestations in humans [9]. This review aims to explore the increase in known and emerging biotoxins in the marine ecosystem, a phenomenon exacerbated by climate change and potentially impacting global life for future generations.

## 2. Eutrophication and Harmful Algal Blooms

In marine ecosystems, climate change can lead to ocean warming, acidification, stratification, and sea level rise. Not only the surface temperature of oceans, but also the inner seas surface temperature is strongly influenced by climate change [10]. Melting ice has increased ocean volume and contributed to an average sea level rise of 3 mm per year, while a total rise of about 40–75 mm by 2100 have been projected. Ocean acidification, resulting from increasing CO_2_ concentrations in the atmosphere, estimates for 2100 a decrease of 0.3–0.4 pH units [11]. These events may alter salinity, pH, and nutrient status of seawater, favoring the growth of harmful algal blooms (HABs) that produce marine biotoxins [12,13]. Eutrophication and climate variability are potential drivers of HAB expansion and intensification, causing negative impacts on marine ecosystems, fisheries and human health [14]. High densities of microalgae cause water discoloration and turbidity, with fish mortality due to hypoxia, altered homeostasis and passive diffusion through gills and epithelium [15]. Globally, the geographic distribution of phytoplankton is mainly influenced by salinity and seawater temperature, but acidification caused by increased atmospheric CO_2_ can also change the ecology of microalgae by promoting their growth [16,17]. The environmental factors which influence HAB proliferation include light intensity, temperature, salinity, acidification and nutrient concentrations [18].

Global warming has intensified and spread HABs from tropical to temperate zones, such as Canada and the United States [19], Europe [20] and the Mediterranean Sea [21]. Higher sea surface temperatures have been associated with increased HAB growth rates and longer growing seasons [22]. However, the impact of climate change on HABs is highly species-specific, favoring the expansion of some phytoplankton species while reducing suitable habitat for others [23]. Furthermore, in regions where they already grow at their optimal temperature, excessive warming can lead to their decline [24]. High ocean temperatures have been reported to increase HABs in spring and autumn, while a decrease is observed in summer, when surface waters become excessively warm [25]. A negative correlation between sea surface temperature and saxitoxin (STX) levels has been observed in the northern and central costal sites in Tasmania [26]. Statistical models have identified a significant relationship between cold water and STX production in combination with other factors such as chlorophyll concentrations and upwelling winds [27].

Diatoms and dinoflagellates are the main phytoplankton communities involved in HAB proliferation. Diatoms are characterized by silicified cell walls with protective function and, therefore, can thrive in turbulent waters; while dinoflagellates do not resist in such conditions, they are able to move in stratified oligotrophic waters due to their two flagella by which they can migrate vertically through the water column [28,29]. The most common genera of harmful microalgae affecting marine ecosystems and fisheries activities are *Alexandrium*, *Gymnodinium*, *Dinophysis*, and *Pseudo-nitzschia* [30]. The UNESCO Intergovernmental Oceanographic Commission (IOC) has prepared a Taxonomic Reference List of Harmful Microalgae providing detailed information on algal species known or suspected to produce toxins. It reports seven Orders and 23 genera of dinoflagellates (Table 1), while diatoms include only the two genera *Nitzschia* (2 species) and *Pseudo-nitzschia* (29 species) belonging to Phylum Bacillariophyta. The dinoflagellate *Azadinium* and *Amphidoma* genera are included in the family *Amphidomataceae*. The UNESCO-IOC database aims at promoting stability in toxigenic microalgal nomenclature and all names are based on the World Register of Marine Species (WoRMS) [31]. The abundance and distribution of harmful algal species are influenced by their structural complexity, as well as by multiple environmental factors such as temperature, salinity, nutrients, light, and hydrodynamic parameters. Numerous studies have been conducted in the Mediterranean Sea to investigate dinoflagellate dynamics, as it is a closed basin connected to the Atlantic Ocean by the Strait of Gibraltar, frequently crossed by large ships and the resulting ballast water discharges, which can introduce toxic microalgae. Rising water temperatures due to climate change further exacerbate its vulnerability, making it more susceptible to non-native algal species [32,33].

HABs produce marine biotoxins, also called phycotoxins, to deter predators and inhibit competing phytoplankton [34]. They cause more or less severe human intoxications, known as paralytic shellfish poisoning (PSP), amnesic shellfish poisoning (ASP), and diarrhetic shellfish poisoning (DSP). STX and domoic acid (DA) are responsible for PSP and ASP, respectively, while okadaic acid (OA) and dinophysistoxins (DTXs) are included in the group of DSP toxins [35]. Marine biotoxins can also be classified based on their chemical structure. While STX and DA are hydrophilic, OA, DTXs, azaspiracids (AZAs), pectenotoxins (PTXs) and yessotoxins (YTXs) are lipophilic compounds. Finally, cyclic polyether brevetoxins (BTXs) are amphiphilic, meaning they exhibit both hydrophilic and lipophilic properties [36,37]. BTXs, responsible for neurologic shellfish poisoning (NSP), are considered emerging marine biotoxins because they have been recently identified but are not yet regulated. Cyclic imines (CIs) are another group of phycotoxins, that include spirolides (SPXs), gymnodimines (GYMs), pinnatoxins (PnTXs) and pteriatoxins (PtTXs), as well as further unknown compounds capable of causing mortality in experimental animals, such as mice, using mouse bioassay (MBA) [38].

Filter-feeding bivalve molluscs, such as clams, oysters, and mussels, can accumulate marine biotoxins and, therefore, are the major source of human exposure. Pursuant to Regulation (EU) 2019/627 [39], the competent authorities shall periodically verify the presence of toxin-producing plankton in production and relaying waters, and marine biotoxins in live bivalve molluscs. Sampling frequency should be weekly during harvest periods, but may be reduced if a very low risk of toxic events can be assumed. Conversely, when changes in toxic algal populations are detected, sampling frequency should be increased and/or precautionary closures of interested areas should be ordered. STX is determined by high-performance liquid chromatography with fluorescence detection (HPLC-FLD) (EN 14526:2026) [40] or any other internationally recognized validated method not using animals, while DA is analyzed by HPLC with ultraviolet detection (UV), although the enzyme-linked immunosorbent assay (ELISA) method may also be used for screening purposes [41]. Lipophilic marine biotoxins (i.e., OA, DTXs, YTXs, and AZAs) are detected by liquid chromatography-mass spectrometry/mass spectrometry (LC-MS/MS) [42]. Finally, the identification of new or emerging toxins, often unknown, is carried out performing MBA or other chemical and alternative methods [39].

### 2.1. Pre-Existing and Regulated Marine Biotoxins

STX and its analogues, such as neosaxitoxin, decarbamoylsaxitoxin and gonyautoxin, are produced by dinoflagellates of genus *Alexandrium* and also by other species (*Pyrodinium bahamense*, *Gymnodinium catenatum* and *Centrodinium punctatum*) [43]. The toxic effects are associated with PSP, characterized by the blockade of voltage-gated sodium channels, with symptoms primarily affecting the nervous system, such as oral paresthesia, visual disturbances, muscle paralysis, and myalgia. They can appear within 0.5–2 h of ingesting contaminated bivalve molluscs and, in severe cases, can lead to respiratory paralysis and death [44]. STX-producing species are distributed around the world, from North America to Australia, Southern Asia, China, Japan, Indonesia, as well as Europe [19]. Severe PSP events have been reported in Spain, Portugal and United Kingdom. As a consequence, the first European monitoring programs aiming at protecting human health and safeguarding the shellfish industry were established [20].

DA is produced by diatoms belonging to the genera *Nitzschia*, *Pseudo-nitzschia*, and *Amphora* [45]. It is an excitatory dicarboxylic amino acid capable of continuously stimulating neurons, causing an increase in intracellular calcium to very toxic levels, resulting in necrosis of neuronal cells in the hippocampus. It is responsible for ASP, which causes dizziness, disorientation, and memory loss, with potentially serious consequences such as coma and, in extreme cases, death. Some gastrointestinal disorders, such as nausea, vomiting, abdominal cramps, and diarrhea, have also been reported [46]. The algal species responsible for ASP have been detected in many temperate regions such as Canada, New Zealand, the Californian coast, and several European countries [19].

OA and DTX are produced by marine dinoflagellates belonging to the genera *Dinophysis* and *Prorocentrum* and cause DSP, which is characterized by gastrointestinal symptoms, such as nausea, vomiting, and diarrhea, but is not considered lethal [47]. They are frequently detected in the regions surrounding the Mediterranean Sea, even at concentrations above the regulatory limits [21]. Mussel samples farmed along the Adriatic coast, central Italy, were analyzed for lipophilic marine biotoxins: the presence of OA and YTXs was detected at levels ranging from 41.6 to 269 μg/kg and from 0.060 to 0.284 mg/kg, respectively. Some samples resulted to be non-compliant for OA concentrations exceeding the maximum limits [35].

AZAs were initially included in the DSP group, but the characterization of their chemical structure and mechanism of action suggested their classification as a stand-alone group [43]. They are produced by planktonic species of genera *Azadinium* (*Azadinium spinosum*, *Azadinium poporum* and *Azadinium dexteroporum*) and *Amphidoma* (*Amphidoma languida*) [48] and are responsible for azaspiracid shellfish poisoning (AZP) showing DSP-like symptoms, but also headache, dizziness, weakness, confusion, seizures, and cardiovascular effects such as hypotension and bradycardia [37]. AZP events have been reported worldwide (North and South America, Africa, Asia and Europe) [49]. YTXs are lipophilic biotoxins produced by dinoflagellates (*Protoceratium reticulatum*, *Lingulodinium polyedrum*, and *Gonyaulax spinifera*) that do not show toxic effects in humans, but have been demonstrated to be cytotoxic and potentially cardiotoxic to rats [49].

In the European Union (EU), Regulation (EC) 853/2004 established maximum limits for marine biotoxins in live bivalve molluscs, corresponding to 800 µg/kg for STX, 160 µg/kg for OA, DTX and AZA, and 20 and 3.75 mg/kg for DA and YTX, respectively [50]. PTX limits were previously included in the OA-group, but were later removed as no adverse effects have been reported in humans [51]. The same safety limits are applied by the Codex Alimentarius, with the addition of the maximum value for BTX (200 mouse units or equivalent/kg) [52]. Similarly, the United States Food and Drug Administration (USFDA) and the Interstate Shellfish Sanitation Conference established a National Shellfish Sanitation Program for the sanitary control of shellfish for human consumption. Under this program, representative water samples and bivalve molluscs must be collected and analyzed for the presence of toxin-producing algae and marine biotoxins, respectively. The criteria for PSP, ASP, DSP and AZP are the same as those established in the EU, while for BTXs they correspond to 200 mouse units/kg of edible portion of raw shellfish or 5000 cell counts for *K. brevis* per liter in the water sample [53].

Of particular concern is the detection of biotoxins in marine organisms other than those commonly known to accumulate, namely bivalve molluscs, which are regularly monitored. In general, it can be assumed that small planktivorous filter-feeding fish, as well as fish feeding on bivalves that host large amounts of marine biotoxins, can enter the food web during toxic algal blooms [54]. For instance, DA has been identified in the guts of lobsters, crabs, and some pelagic fish such as sardines, anchovies, mackerel and tuna along the West coast of the United States, as well as mullet, pompano, kingfish in the Gulf of Mexico. Similarly, PSP toxins have been found in gastropods, echinoderms, and cephalopods, posing a significant risk to humans if harvested from contaminated waters [53]. Lipophilic toxins have been detected in different invertebrate benthic organisms, such as echinoderms, arthropods and non-traditional molluscs, as new vectors of these toxins [55], while BTXs have been found in fish (especially prey species like sharks or rays), crustaceans and marine mammals (dolphins, manatees), causing their massive death in Florida between 1999 and 2006 [56]. These studies highlight that particular attention should be paid to alternative hosts that are not yet fully integrated into public health surveillance systems.

### 2.2. Marine Biotoxins Not Yet Regulated

BTXs are lipid-soluble and heat-stable cyclic polyether compounds, including several metabolites (brevisamides, brevisins, brevenals, tamulamides, etc.) produced by the dinoflagellate *Karenia brevis* and, when ingested through the consumption of bivalve molluscs such as mussels, oysters, clams and scallops, are responsible for NSP. They have a ladder-like structure similar to CTX, characterized by a specific affinity for voltage-gated sodium channels [57]. Symptomatology includes nausea, vomiting, diarrhea, paresthesia, ataxia, bradycardia, dizziness, loss of coordination and coma in more severe cases [58]. BTX exposure in humans may also occur through inhalation or skin contact with contaminated water, causing symptoms in the respiratory system (e.g., coughing, sneezing, rhinorrhea, and a burning sensation in the noise and throat) and skin irritation. Inhaled aerosolized BTXs can also cause respiratory irritation in healthy individuals and asthma exacerbation in affected subjects. Inflammatory and immune effects have been reported due to BTX, such as recruitment or activation of immune cells, as well as systemic inflammatory response [59]. BTXs have been reported in the Atlantic Ocean from the Gulf of Mexico to Florida, as well as in the West Indies and New Zealand. Recently, they have been detected in French Mediterranean mussels from Corsica, at maximum concentrations of 345 µg/kg in the digestive gland, corresponding to an estimated value of 57 μg/kg of total flesh [60].

Palytoxins (PLTXs) and ovatoxins (OVTXs) are atypical marine biotoxins that cause intoxication in humans through multiple exposure routes (diet, inhalation, and skin). They are produced by the genus *Ostreopsis*, initially present in tropical regions, but which has also spread to temperate latitudes, particularly the Mediterranean Sea [61]. PLTXs were first isolated from the tropical soft coral *Palythoa toxica* in Hawaii, but can also be present in fish, crabs, and other marine invertebrates, as well as in zoanthids that may be found in home and commercial aquariums. Dermal, ocular, and respiratory exposures have been described in swimmers, divers, or fishermen near waters containing *Ostreopsis* blooms, as well as in marine aquarium hobbyists, while presence in seafood can be recognized by an unusual metallic taste [62]. Symptoms of PLTX poisoning include nausea, vomiting, diarrhea, dizziness, numbness of the limbs, muscle cramps, paresthesia, difficulty breathing, bradycardia, and death in more severe cases, while OVTX intoxication is characterized by flu-like symptoms, eye irritation, and respiratory distress [63]. They have been documented in both temperate and subtropical regions in the Mediterranean Sea as well as the Atlantic Ocean, Pacific Ocean and the Caribbean region [32].

CIs constitute a new group of marine biotoxins with no proven acute toxicity to humans, but their presence is increasing in warming ocean environments. In addition to the four major subgroups previously mentioned, CIs include other lipophilic compounds which share a cyclic imine moiety in their structure, such as prorocentrolide, spiro-prorocentrimine, portimine, and symbioimine [64]. CIs were discovered accidentally by MBA on shellfish extracts used for NSP/DSP monitoring purposes, causing unusually short death times in mice due to interactions with nicotinic acetylcholine receptors at the neuromuscular junctions [65].

SPXs are produced by *Alexandrium* spp. (*Alexandrium ostenfeldii* and *Alexandrium peruvianum*) and are globally distributed along the European, North and South American waters, while *Karenia selliformis* and *Vulcanodinium rugosum* are the main producers of GYMs and PnTXs, respectively. The names PnTX and PtTX are derived from the algae species from which they were first isolated, i.e., *Pinna attenuata* and *Pteria penguin*, respectively [66]. GYMs were initially detected in New Zealand, but later also in Europe, Morocco, Lebanon, South Africa, Australia, China and Tunisia, while SPXs were isolated in Canada and have spread to Argentina, Europe, China and New Zealand. PnTXs and PtTXs were first discovered in China and Japan, respectively [64,66]. As there have been no cases of human poisoning following consumption of contaminated seafood, CIs are not yet regulated in the EU, although the exposure to these biotoxins continues to increase, due to rapid changes in marine ecosystems caused by global warming and rising ocean temperatures, and the development of analytical methods for their detection have been recommended [67]. The application of LC-MS/MS to the analysis of GYMs, SPXs, PnTXs and PtTXs in mussels from the Galicia, north-western Spain, revealed PnTX and SPX concentrations ranging from 1.8 to 3.1 μg/kg and from 1.2 to 6.9 μg/kg, respectively [68]. SPX and GYM levels up to 29.2 and 12.1 μg/kg have been found in mussels from north–central Adriatic Sea (Italy), respectively [66].

According to the CLEFSA project, some marine biotoxins (DA, OA, PLTXs, PnTXs, TTXs and CTXs) are considered emerging issues, defined as those arising from a new identified hazard or increased exposure/susceptibility to a known hazard. Because climate change may increase the severity, duration, and frequency of potential impacts of these identified issues, applying forward-looking approaches that broaden the time horizon of the investigation from the short to the long term and adopting a more systemic view of the food supply chain represents a valuable tool. Some recommendations to future food safety challenges are based on continuous monitoring and data collection, as well as a detailed risk characterization by a specific network of experts, namely the Emerging Chemical Risks Identification Working Group [69]. Regarding marine biotoxins belonging to the CI group, the occurrence data obtained from literature demonstrated low SPX concentrations and thus a low risk for consumers, while GYMs and PnTXs have been occasionally detected at higher levels, but no related illnesses have been reported. Nevertheless, monitoring studies are needed to better understand their chronic effects [70]. Table 2 summarizes the symptomatology of some marine biotoxins on human health.

## 3. Poisonous Fish Containing Neurotoxic Marine Biotoxins

While the above marine biotoxins are mainly detected in live bivalve molluscs as filter-feeding organisms, the main sources of human exposure to ciguatoxins (CTXs) and tetrodotoxins (TTXs) are fish belonging to specific families or living in contaminated areas. According to Regulation (EC) 853/2004, fish products derived from poisonous fish known to contain TTXs and belonging to the following families *Tetraodontidae*, *Molidae*, *Diodontidae* and *Canthigasteridae*, or from fish containing biotoxins such as CTXs or muscle-paralyzing toxins, must not be placed on the market for human consumption [50]. TTXs have recently been detected not only in poisonous fish, but also in bivalve molluscs and marine gastropods in European waters [71], while CTX presence in various marine organisms, such as giant clams and sea urchin, has been reported [72,73].

### 3.1. Ciguatoxin Origin and Toxicity

Ciguatera Fish Poisoning (CFP) is generally caused by the consumption of tropical coral reef fish contaminated with CTX, which is a neurotoxin produced by benthic dinoflagellates belonging to the genera *Gambierdiscus* (19 species) and *Fukuyoa* (4 species) [74]. CTX can accumulate in herbivorous and detritivorous fish that feed directly on contaminated algae, and further in piscivorous fish through the food web [75]. More than 400 fish species are considered potential CTX vectors, belonging to the families *Sphyraenidae*, *Serranidae*, *Lutjanidae*, *Carangidae*, *Labridae*, *Scombridae*, *Acanthuridae*, and *Muraenidae* [76]. Among herbivorous species, the most commercially known are the surgeonfish and the parrotfish, while carnivorous fish include the moray eel, grouper, red snapper, dusky grouper, barracuda, amberjack, and trevally. Significant interspecific differences in CTX concentrations have been reported in different fish species, due to the higher toxin content and greater bioaccumulation capacity of higher trophic level fish. Furthermore, less or more oxidized CTX forms (the latter being more toxic) are found in fish of lower and higher trophic levels, respectively. However, because CTXs are tasteless, colorless, and odorless, it is very difficult to distinguish the fish that cause CFP [77].

The CTX group includes other cyclic polyether toxins called maitotoxin (MTX), gambieric acid, gambierol, gambieroxide, and gambierone [78]. The mechanism of action is closely related to their chemical structure. CTX and gambierone activate the voltage-gated sodium channels, causing their persistent activation and increased neuronal excitability. As CTXs share a common binding site on sodium channels with the structurally similar polyether BTX, CFP is comparable to NSP [79]. Conversely, MTX and its analogues are calcium channel antagonists, resulting in a massive influx of calcium and rapid cell death [80]. The presence of at least one sulfate group in MTXs is responsible for increased polarity, which limits their adsorption when ingested. Additionally, since they are mainly present in the digestive tract and liver of fish, they do not accumulate in the meat and, therefore, rarely cause CFP [78]. Gambierol acts primarily by blocking voltage-gated potassium channels [81].

CFP was initially restricted to tropical and subtropical areas at latitudes 35° N and 35° S (Pacific and Indian Oceans and Caribbean Sea), but has actually expanded into temperate waters as well, mainly due to the proliferation of HABs as a consequence of ocean warming and climate change [82]. Recent studies have reported its presence in non-endemic regions such as Korea, Japan, the Canary Islands, the Mediterranean Sea, the eastern coasts of North and South America, and the northern Gulf of Mexico [83]. It is initially characterized by gastrointestinal symptoms (diarrhea, vomiting, and abdominal pain), which appear after 12 h and resolve within 24–48 h. More rarely, musculoskeletal (weakness and fatigue), and cardiovascular (hypotension, bradycardia) symptoms may occur, but the typical neurological symptoms are paresthesias and cold allodynia, commonly reported as temperature inversion and considered patognomonic [84,85]. CFP can also cause long-term effects that persist for days, months, or even years and, in severe cases, can lead to paralysis, coma, and death [86]. It is a notifiable disease in Queensland, eastern Australia, where most cases are caused by fish caught from the Great Barrier Reef. However, it is often underestimated because patients do not associate their symptoms, which are often mild, with fish consumption [87].

It is important to note that CFP can present symptoms similar to other marine biotoxin intoxications with neurological effects, such as PSP, NSP, and TTX. Specifically, since CTXs show a similar mechanism of action to BTXs, cold allodynia can also be observed in patients with NSP, although no cases have yet been documented after fish consumption [88]. As previously reported, BTX is found mainly in bivalve molluscs, and contamination of fish is a rare event [89]. However, there are differences in potency between BTX and CTX, as the former acts at micromolar concentrations, while CTX exerts its effects at nanomolar concentrations. Furthermore, CFP symptoms are more severe and longer-lasting compared to NSP, which can be considered a mild form with similar but less severe symptoms [90]. Guidance levels of 0.1 and 800 µg/kg have been established by the USFDA for CTX and BTX, respectively [76], while in the EU no regulatory limits are set for these two groups of biotoxins, and the marketing of fish suspected of being contaminated with CTX is prohibited [50].

### 3.2. Tetrodotoxin in Poisonous Fish Families and Other Marine Organisms

TTX is a potent neurotoxin found primarily in pufferfish, which in Japan is consumed as a highly prized delicacy called Fugu, but its preparation requires specialized training for Japanese chefs and a regulatory limit of 2 mg TTX/kg of pufferfish tissue that can be consumed without adverse effects on humans has been established [91,92]. The name TTX is derived from the family *Tetraodontidae*, which includes many genera known to contain this toxin, such as *Sphoeroides*, *Lagocephalus*, *Arothron*, *Amblyrhynchotes*, *Chelonodon*, and *Dichotomyctere* [93]. TTX can be present in many parts of the body of a fish, particularly the skin, liver, gonads, intestines, salivary glands, but this distribution can vary depending on habitat, season, and lifespan. For example, in the autumn–winter period, it is mainly present in the ovaries during maturation, while after spawning in the spring–summer period, it is more concentrated in the liver and/or skin [94]. The origin of TTX in fish is still controversial. Some authors reported the association with specific dinoflagellates of genera *Alexandrium* and *Prorocentrum* [95,96]. Conversely, other studies in the literature have demonstrated the presence of this toxin in several microbial strains belonging mainly to the genera *Vibrio*, *Aeromonas*, *Alteromonas*, *Pseudomonas*, *Shewanella*, but also representative of the genera *Bacillus*, *Alcaligenes*, *Enterococcus*, *Micrococcus*, etc. Detection in marine sediments has suggested its bioaccumulation through small zooplankton and detritivorous organisms along the food chain [97].

Although TTX poisoning is mainly reported in Asia due to the consumption of contaminated fish, TTX presence has been recently reported in edible marine species in subtropical and temperate waters around Europe [98]. The extent of this phenomenon is well represented in the Mediterranean Sea, where tropicalization and global warming have favored the entry of Indo-Pacific fish species through the Suez Canal. Guardone et al. [99] reported the presence of fish belonging to the *Tetraodontidae* family along the Italian coasts and, specifically, *Lagocephalus sceleratus* (110,237 individuals since 2003), *Lagocephalus lagocephalus* (126 and 110 specimens between 1878 and 2003) and *Sphoeroides pachygaster* (716 and 104 from 1979 to 2003). The spread of *L. sceleratus* in the Mediterranean Sea has been estimated by using machine learning algorithms describing a generally decreasing impact along the western coasts. Conversely, high-risk areas have been predicted in some central and southern zones such as Sicily, Malta, and Tunisia [100]. The EU legislation prohibits the marketing of pufferfish and other species belonging to specific poisonous fish families [50].

Over the past two decades, the number of reports of TTX-contaminated marine shellfish has increased both in the Northern and Southern hemispheres [101,102]. A potential role of increased seawater pH and temperature caused by climate change on the growth of TTX-producing microorganisms, such as *Vibrio*, *Shewanella* and microbial strains of *Flavobacteriaceae* family, has been recently described [103,104].

TTX acts by selectively blocking the voltage-gated sodium channels, resulting in the inhibition of depolarization and conduction of action potentials in nerve and muscle cells [105]. The initial symptoms include perioral tingling, headache, sweating, muscle weakness, followed by complications such as hypotension, arrhythmia, ataxia, muscle paralysis, and death due to respiratory failure [106]. Although TTX and STX show different chemical structures, their mechanism of action is comparable. They both bind to site 1 of voltage-gated sodium channels blocking the sodium ion influx into the cells [105]. They also contain functional chemical groups that are affected by pH, and acidification due to climate change can exacerbate their toxicity. Indeed, it is known that their protonated forms, more prevalent in acidic conditions, exhibit stronger electrostatic interactions with ion channels, resulting in complete blockage of voltage-gated sodium channels in nerves and muscles [107]. Furthermore, STX is more stable under acidic conditions (pH < 4), whereas with increasing pH, the guanidine group donates hydronium ions, resulting in a decrease in its charge value and potentially altering its interaction with sodium channels [44]. CTXs and BTXs exhibit an opposite mechanism of action by binding to site 5 of voltage-gated sodium channels and slowing the inactivation of the sodium ion current, resulting in the exacerbation of excitability [108].

## 4. Challenge and Future Perspectives

Recent and accelerated climate change phenomena have exacerbated the presence of toxin-producing algal species in marine ecosystems worldwide. Future scenarios predict significant and increasing risks regarding all affected areas (water, food, marine ecosystems, and humans). The serious, sometimes even fatal, effects on human health underscore the need for appropriate policy measures and interventions to mitigate the environmental risks posed by climate change globally. The urgency of addressing the increasing frequency and intensity of extreme weather events is evident, as more and more countries face the impacts of global warming and sea level rise. The UNESCO Intergovernmental Oceanographic Commission (IOC) has launched a global literature search project to create a reliable and verified database of all toxic algal taxa, categorized by geographic region. The goal is to provide training and expertise in species identification, understanding the causes of this phenomenon, predicting its occurrence, and potentially mitigating its effects. The Ocean Biodiversity Information System (OBIS) gives biogeographical information on microalgae species through reference maps, while HAEDAT (Harmful Algal Event Database) is the only existing open-access database containing information on HAB events worldwide [109]. Specifically, it provides data on water discoloration events, precautionary closures of harvesting areas due to the presence of toxic algae, non-compliant seafood based on the levels of marine biotoxins, and any incidents in which humans, animals, or other organisms have been adversely affected [21].

Several statistical and process-based approaches have been proposed to link global climate model projections and potential HAB responses. Such approaches often require decades of HAB monitoring, which is lacking in many affected regions. Furthermore, when based on theory or laboratory studies, they do not always account for all variables that can occur naturally and which can lead to uncertainty in model projections. Climate change response models depend on the ability to predict forcing conditions, such as water temperature, wind speed, and other geochemical factors which can influence HAB response [110]. For instance, recent studies have assessed the impact of four abiotic predictors (sea surface temperature, salinity, current velocity, and bathymetry) on the distribution of three different PSP dinoflagellate species, demonstrating an increasing habitat suitability in the Northern Hemisphere and a decreasing suitability in tropical areas [111]. Reducing GHG emissions, strengthening early warning systems (EWSs) to forecast HAB events, promoting international collaboration, and raising public awareness are crucial implementation tools [1]. EWGs have been developed to identify and predict HAB behavior in a specific geographic area. They can be based on bulletin reports of phytoplankton and/or marine biotoxin monitoring datasets or particle tracking-based systems to predict their transport. Further EWGs utilize statistical models which analyze the relationships between environmental factors and the development of toxic events. Online monitoring systems are capable of automatically tracking phytoplankton and water quality parameters (temperature, turbidity, pH, dissolved oxygen, etc.) that can be used through process-based models which analyze the physics, chemistry, and biology of HAB formation [112]. Prevention policies for managing HABs generally include monitoring programs and ecosystem-based management, reducing nutrient inputs or creating green infrastructure to capture nutrients before reaching waterways. Mitigation practices can be based on physical methods, such as cleaning operations or clay flocculation, or biological and chemical strategies through the use of algicidal bacteria and hydrogen peroxide, respectively. Further actions include shellfish harvesting bans and regulatory limits for marine biotoxins in seafood [113].

## 5. Conclusions

Proactive measures across multiple sectors could mitigate the growing risk of climate-related toxicological threats to future generations by adopting an ecologically relevant perspective within a One Health approach [114]. Regulators and public health authorities should strengthen environmental policies to regulate emerging toxins and implement early warning systems for their detection. The industrial sector should adopt sustainable practices to reduce polluting sources and environmental waste, since greenhouse gas emissions from human activities contributed to a warming of 1.0 °C to 2.0 °C [2]. Finally, scientists could develop interdisciplinary approaches that integrate toxicology, climate science, and public health, ensuring that communities and populations receive priority information on human toxicological alerts.

## Figures and Tables

**Table 1:** Orders and genera of dinoflagellates according to the UNESCO Intergovernmental Oceanographic Commission.

Orders	Genera (Number of Species)
Amphidiniales	*Amphidinium* (6)
Dinophysales	*Dinophysis* (10) *Phalacroma* (1)
Gonyaulacales	*Alexandrium* (17) *Centrodinium* (1) *Coolia* (4) *Fukuyoa* (3) *Gambierdiscus* (17) *Gonyaulax* (4) *Lingulaulax* (1) *Ostreopsis* (6) *Protoceratium* (1) *Pyrodinium* (1)
Gymnodiniales	*Akashiwo* (1) *Gymnodinium* (1) *Karenia* (9) *Karlodinium* (7) *Mergalefidinium* (2) *Polykrikos* (1)
Peridiniales	*Heterocapsa* (5) *Vulcanodinium* (1)
Prorocentrales	*Prorocentrum* (15)
Thoracosphaerales	*Pfiesteria* (2)

**Table 2:** Marine biotoxins causing toxic effects on humans.

Toxin	Gastrointestinal Symptoms	Neurological Symptoms	Other Symptoms
STX	Abdominal pain, nausea, vomiting, diarrhea	Tingling, numbness, burning of the mouth and lips, ataxia, muscle weakness, staggering, loss of coordination, giddiness, drowsiness	Fever, rash, headache, irregular heartbeat, difficulty breathing, respiratory paralysis and death
DA	Nausea, vomiting, abdominal cramps, diarrhea	Confusion and disorientation, seizures, hallucinations, dizziness, short-term memory loss, coma and death	Headache, respiratory secretions, profuse respiratory difficulty, cardiac arrhythmias
OA	Nausea, vomiting, abdominal cramps, diarrhea	Dizziness, seizures, hallucinations, short-term memory loss	Chills, fever, headache; impairs immune system function
AZA	Nausea, vomiting, diarrhea, stomach cramps	Not observed	Not observed
BTX	Nausea, abdominal cramps	Weakness and difficulty of movement, paralysis, convulsions, coma and death	Coughing, asthma-like symptoms, wheezing, shortness of breath, chest tightness

## Data Availability

No new data were created or analyzed in this study. Data sharing is not applicable to this article.
